# The pitfalls of ectomycorrhizal microcosms: lessons learnt for future success

**DOI:** 10.1080/15592324.2025.2527378

**Published:** 2025-07-07

**Authors:** André Geremia Parise, Vinicius Henrique De Oliveira, Mark Tibbett, Brian John Pickles

**Affiliations:** a School of Biological Sciences, University of Reading, Reading, UK; b School of Agriculture, Policy and Development & Soil Research Centre, University of Reading, Reading, UK

**Keywords:** Hyphae, maze, microcosm, negative results, *Pinus sylvestris*, seedling, structural information, *suillus granulatus*

## Abstract

Mycorrhizal fungi are known to support their host plants by facilitating nutrient acquisition and enhancing resistance to biotic and abiotic stress. However, the possibility that they also convey structural information about the soil has not yet been tested. Here, we attempted to investigate whether ectomycorrhizal hyphae could guide root growth in response to physical obstacles by using Scots pine (*Pinus sylvestris*) and *Suillus granulatus* in a microcosm experiment fitted with U-shaped silicone mazes. Despite initial success in achieving ectomycorrhizal colonisation (88% of the inoculated seedlings), the fungi failed to produce the expected hyphal networks. Extensive and unexpected root growth rendered the system unsuitable for testing our hypothesis. Furthermore, structural issues with the microcosms compromised substrate integrity, possibly inhibiting fungal development. While our results were inconclusive, this report highlights challenges associated with replicating classical ectomycorrhizal experiments, underscoring the need for methodological refinement. We provide detailed recommendations and methodological clarifications that may aid future research. Although our initial hypothesis could not be tested, we argue that traditional microcosm experiments retain potential for advancing our understanding of mycorrhizal ecology, provided they are critically revisited and technically improved. Negative results, when well contextualised, are valuable contributions toward more robust and reproducible experimental frameworks.

## Introduction

Over a hundred years of research into mycorrhizal symbioses have elucidated many roles for this interaction between plants and their associated fungi. Several studies have shown that, when in association, mycorrhizas increase plant nutritional status,^1–3^ protect plants from pathogens and diseases,^4–6^ improve resistance to abiotic stress,^7,8^ and potentially help seedling establishment.^9–11^ However, many questions remain open, and the full implications of this symbiosis for both plants and fungi are far from being entirely known.

One aspect of the symbiosis that has hitherto been ignored is whether, beyond providing nutrients and water to their host plants, mycorrhizal fungi might also provide the host with information about the structure of the belowground environment. It is known that many trees delegate their foraging behaviour to mycorrhizal fungi.^2,12,13^ Instead of growing roots to seek and exploit nutrient patches, they employ the more versatile, dynamic, and carbon-efficient mycelial systems.^14–16^ This process is known as foraging complementarity,^13^ and seems to be more present in tree species with thick (i.e., ~ > 0.4 mm-wide) absorbing roots.^12,17^ However, if fungal hyphae are growing beyond the roots, scouting ahead of them, they may find structural complexities in the soil, like rocks or zones of compaction, and divert away from them, eventually guiding root growth to avoid these obstacles. To our knowledge, this hypothesis has never been explicitly tested.

Here, we carried out an experiment to test the hypothesis that the growth of mycorrhizal hyphae could provide structural information to the host plant about the belowground environment. We used Scots pine (*Pinus sylvestris* L., Pinaceae) and the ectomycorrhizal fungus *Suillus granulatus* (L.) Roussel (1796) (Boletaceae). The fungus was chosen because, like others in its genus, it is easy to grow in axenic culture, it associates easily with hosts under experimental conditions, and it produces large hyphal strands that are visible to the naked eye.^18^ The plant species was chosen because it is an ectomycorrhizal host with thick roots (i.e., its root tips are usually 0.47–0.48 mm thick, see ^19^ and,^20^ and therefore likely to depend on its fungal partner to explore the environment. Scots pine is native throughout the mountainous boreal regions of Eurasia, from Scotland to Siberia,^21^ where the soil is often rocky and potentially challenging to navigate. A young seedling that has just germinated from a small seed must grow its roots into suitable areas and avoid dead ends and cracks between the rocks. We infer that the metabolic cost for a small seedling to correct this growth is likely to be high. We hypothesised that ectomycorrhizal fungi could help the seedling mitigate its carbon costs, potentially leading to more carbon available for the fungal partner, by guiding its roots to the most suitable soil patches for stability and further growth.

To carry out this study, we attempted to replicate some classical experiments on ectomycorrhizas using *Pinus sylvestris* and *Suillus* spp.^22–25^ We compared papers from the literature that conducted experiments with pine seedlings to understand the methods used, and tried to follow them. Our intention was to grow inoculated *P. sylvestris* seedlings in thin Perspex microcosms that would allow the observation of root and hyphal development. An obstruction in the soil was simulated by affixing a U-shaped silicone maze placed below the seedling. We predicted that the fungal hyphae would grow faster than the roots, reach the bottom of the maze, and potentially signal to the plants that an obstacle was present, which would trigger more lateral root formation as a response to avoid the maze. Consequently, we would expect more root mass inside the maze for plants that are not inoculated with *S. granulatus* than for plants that were inoculated, which would have more lateral root development to avoid the maze. This kind of maze was chosen because it was also used in other experiments with slime molds, organisms with a similar structure and behaviour as fungi,^26^ and to study the behaviour of arbuscular mycorrhizal hyphae.^27^ It was also used in tests with simple robots to test the robots’ ability to escape basic traps like a dead end.^28,29^

## Materials and methods

### Synthesis of mycorrhizas

The techniques described here were inspired by works like Duddridge, Finlay and Read, Rosling et al., and Bending and Read.^22–25^ *Pinus sylvestris* seeds were acquired from Chiltern Seeds (Wallingford, UK). The seeds were harvested in plantations in Shropshire and Norfolk (UK) between 2019 and 2020, and had been stored at −4 °C until purchased in March 2023, subsequently being stored at 4 °C until sown. To obtain aseptic seedlings, the seeds were surface sterilised in a laminar flow cabinet by soaking them in H_2_O_2_ 30 % for 15 min in a glass beaker, stirring often to ensure sterilisation. Then, the H_2_O_2_ was removed with a pipette and the seeds were washed 5 times with autoclaved milli-Q water. After the fifth wash, the seeds were covered with autoclaved milli-Q water, and the beaker was closed with aluminum foil and kept in the dark, refrigerated at 5.5 ± 1 for 72 h. Then, again in the laminar flow cabinet, the seeds were sown in Petri dishes with agar (15 g · L^−1^) and glucose (2 g · L^−1^), sealed with Parafilm®, and taken to a 2.50 × 1.85 ×2.00 (W x L x H) controlled environment room (Fitotron® SGR – Weiss Technik, Heuchelheim, Germany). The Petri dishes were kept tilted at approximately 45 ° in a 16 h daylight regime (06:00–22:00), 15 °C during the day and 10 °C during the night, humidity constant at 60 %, and photon flux density 170 µmol m^2^ · s^−1^ PAR. This procedure was based on information retrieved from the articles cited above, and a comparison between the methods for synthesising mycorrhizas can be found in Supplementary Material 1.

**Figure 1.**
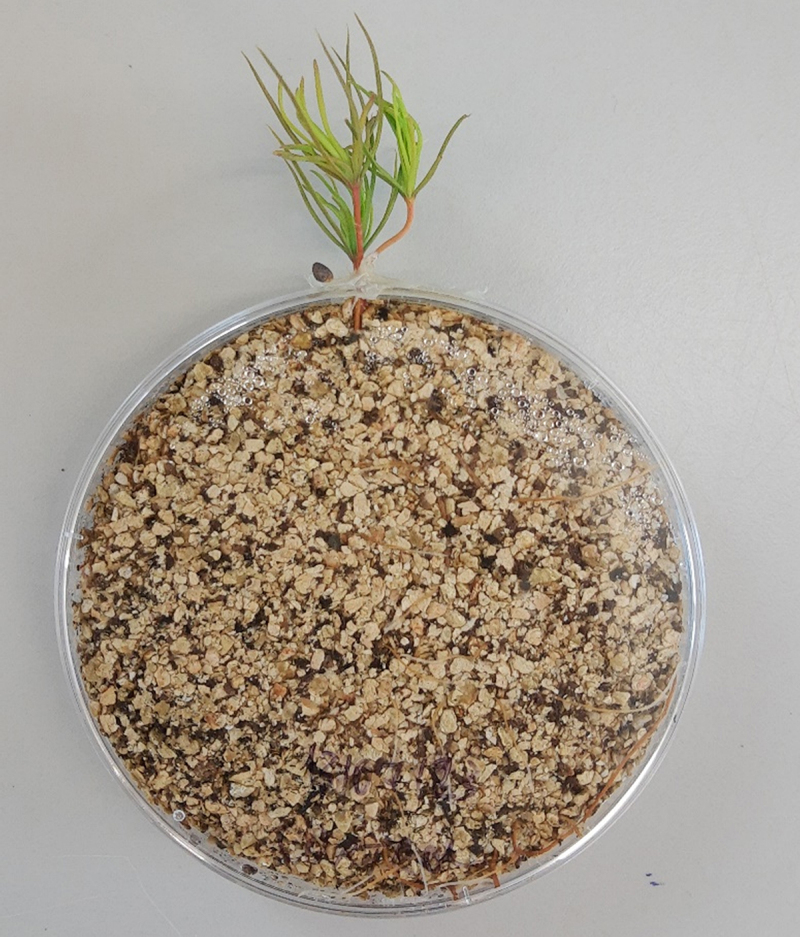
Example of the inoculation set-up. Three *P. sylvestris* seedlings were positioned with the roots inside a petri dish filled with peat and vermiculite, inoculated with agar plugs with *S. granulatus*, and sealed with Parafilm® and anhydrous lanolin.

After 20 days, the seedlings were inoculated with *Suillus granulatus* obtained from the University of Reading mycological collection. In a laminar flow cabinet, Petri dishes were prepared by carving a notch in one of the edges with a hot scalpel. They were filled with peat and vermiculite (1:4, v:v) that had been previously sieved through a 2 mm mesh and disinfested by autoclaving at 105 °C for 1 h on two consecutive days. Three seedlings were laid on the peat with the stems protruding outside through the notch (Figure 1). Two or three agar plugs (⌀ 11 mm) containing the growing edges of a 24-day-old culture of *S. granulatus*, cultured on potato-dextrose-agar (PDA; Thermo Fischer Scientific, Waltham, Massachusetts, USA), were placed onto the root tips. The roots and agar were covered with a layer of the pre-prepared peat and vermiculite and moistened with a liquid Modified Melin-Nokrans nutrient medium (MMN) without a carbon source by spraying approximately 28 mL of medium on it with a spray bottle. This was enough to soak the substrate. Then, the Petri dish was closed and sealed with a Parafilm® strip and anhydrous lanolin around the stems. The control plants underwent the same procedure, but without adding the agar plugs. The Petri dishes were wrapped with aluminum foil, taken to the same controlled environment room and conditions described before and kept vertically. After approximately two days of acclimation, the photon flux density was increased to 210.5 µmol m^2^ s^−1^.

### Setting up the microcosms

**Figure 2.**
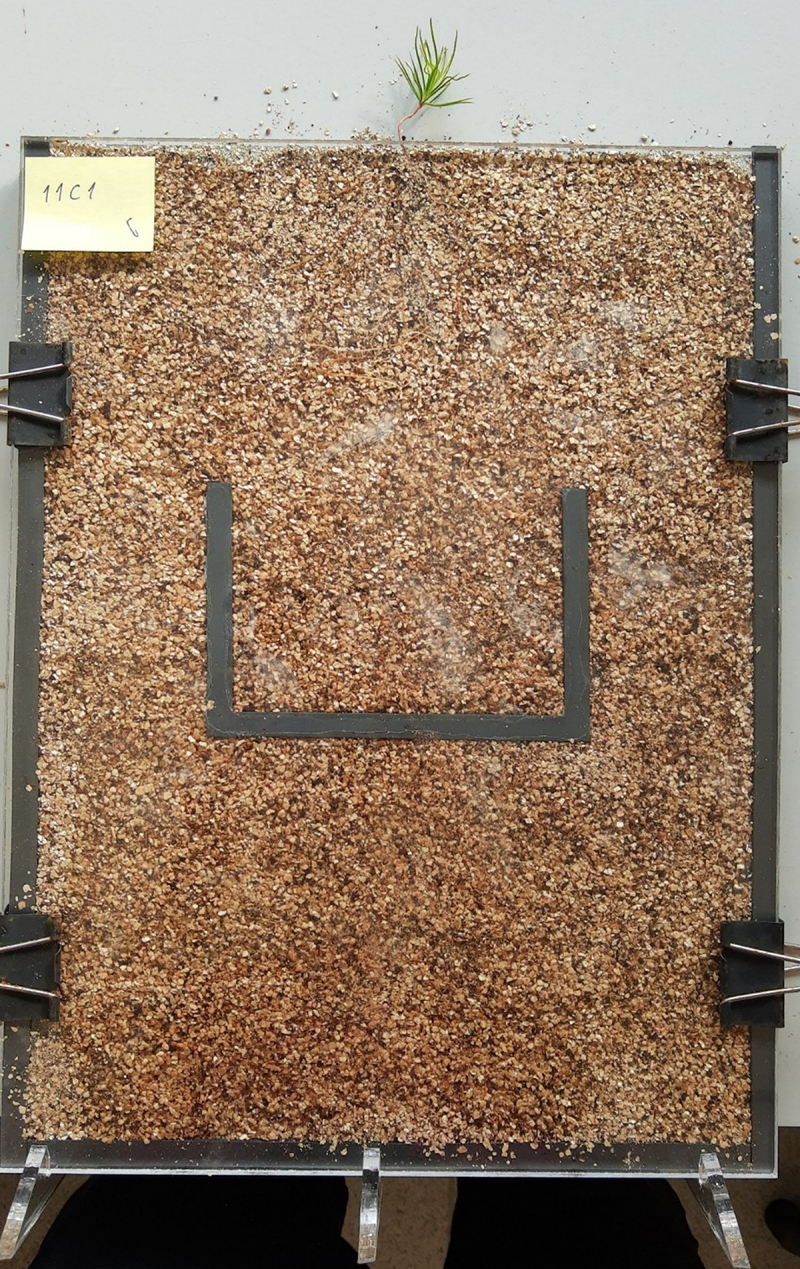
Photograph of one of the microcosms prior to being wrapped in aluminium foil and before the interventions to secure it.

**Figure 3.**
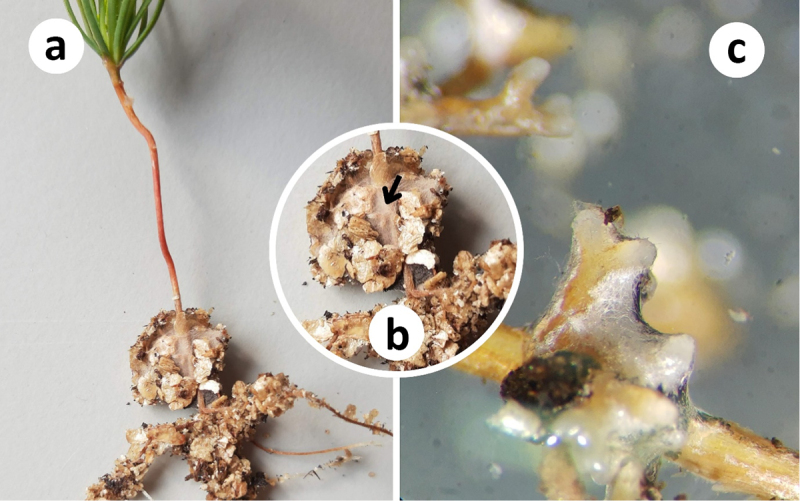
Inoculated seedlings. A: after the inoculation period with *Suillus granulatus*, some seedlings of *Pinus sylvestris* had evident ectomycorrhizas, with the fine root tips well shrouded by the hyphal mantle. B: close-up of the root section of the seedling shown in A exhibiting two root tips (arrow) surrounded by a thick mantle of hyphae. C: root tip of one seedling not used in the microcosm, under the stereomicroscope, showing the development of tip and mantle, with characteristic hydrophobicity.

After 60 days from inoculation, the seedlings were transferred to the microcosms. The microcosms consisted of a pair of 0.6 cm thick 30 × 40 (W x H) Perspex plates separated by 0.3 cm thick and 1 cm wide black silicone spacers. The spacers were glued with the silicone sealant in all the inner edges except for the top of the microcosm. In the middle of the microcosm, a silicone maze shaped like a square U was also glued with silicone sealant (Figure 2). A plan of the microcosm with the position of the maze and all the measures can be found in Supplementary Material 2. Then, the microcosms were filled with the sterile mix of peat and vermiculite, moistened by spraying MMN medium. One seedling was placed on the top of the microcosm. Subsequently, silicone sealant was applied along the maze, and the microcosm was covered with the other Perspex plate. Four 0.41 cm-wide foldback clips were used to hold the plates together. The control and inoculated microcosms were set up alternately by two people to avoid bias between how the experimental groups were set up. Some seedlings had already evident and well-formed mycorrhizal tips before being transferred to the microcosms (Figure 3a,b).

Finally, all the microcosms were wrapped with aluminum foil and taken to the same growth room. Since the seedlings were now at a higher position (40 cm above the bench), they were exposed to approximately 302.1 µmol m^2^ s^−1^ PAR. In total, we set up 15 inoculated and 15 control microcosm. The other environmental conditions remained the same as before.

Inoculated seedlings that were not used for the microcosms were quickly analysed under a stereo microscope to assess colonisation. We checked for the presence of hyphae and fine roots (Figure 3c) as a proxy for the presence of ectomycorrhizas. Then, they were wrapped in moist paper-towel and stored in plastic bags in the fridge at 4 °C for morphological subsequent analysis.

### Fixing problems with microcosms

After a few days, we noticed that the Perspex plate had bent outwards in the extremities, markedly at the upper side, exposing the substrate and the roots. One by one, they were taken out of the growth room and unwrapped. We added four extra foldback clips of the same kind to the extremities. Then, on each side above the maze an extra 30 × 10 cm Perspex plate (0.6 cm thick) was placed and held by two spring clamps with a 5.0 cm opening (Manufacturer ID: T58200EL7. Irwin Industrial Tools, Huntersville, North Carolina, USA). The new Perspex and spring clamps applied, uniformly, more pressure on the mazes. After adding those new components to the microcosms, the dry upper layer of substrate was moistened by spraying milli-Q water, and the microcosm was completed with dry substrate added to the top. The substrate was dry to create an air cushion between the moist substrate below and the atmosphere, hence retaining more water in the microcosms.

Finally, the microcosms were wrapped in new aluminum foil and returned to the growth room. All microcosms were adjusted in this way over 1 week.

### Plant harvest

After four weeks (31 days) since setting up the microcosms, harvest started. We first checked some plants and noticed that they barely grew into the maze, and no developed hyphae were seen. Therefore, we decided to harvest only eight plants and leave the remaining ones for further two weeks in order to check for development of roots and hyphae.

The choice of the plants to be harvested was made using a random number generator website (https://sorteador.com.br). The microcosms were photographed, the plants were wrapped in moist paper-towel and stored in a fridge at approximately 4 °C for later analysis.

### Plant morphology analysis

On the day following the harvest, we washed the roots thoroughly to remove as much substrate as possible. Then, we scanned all roots of the seedlings from the experimental microcosms as well as those not used in the microcosms, using the software WinRHIZO^TM^ (Regent Instruments Inc., Ottawa, ON, Canada). Morphological parameters analysed were total root length (cm), total root area (cm^2^), total root volume (cm^3^), and number of root tips (not necessarily ectomycorrhized root tips).

We took pictures of the microcosm with a Motorola One Action cell phone (Motorola, Inc., Schaumburg, Illinois, USA) and used ImageJ (version 1.54, National Institutes of Health, Bethesda, Maryland, USA) to measure the height of the seedlings. This was done by converting the picture in an 8-bit grayscale image with the command *Image > Type > 8-bit*, then setting the scale for each image using the 1 cm edge of the maze as a reference, and finally measuring the length of the stem from the substrate to the basis of the first needle with the *Segmented line* tool.

### Statistical analysis

All analyses were carried out with the software XLSTAT®. We transformed the data by √x to obtain normality (Shapiro-Wilk, *p* > 0.05), and homoscedasticity was checked with a Levene test (*p* > 0.05). After parametric assumptions were met, we carried out a preliminary three-way ANOVA to verify if there were any influence of two factors: “evidence of ectomycorrhizas prior to transplanting” and “experimenter identity”. Since neither factor was significant (*p* > 0.05), a one-way ANOVA was carried out for all variables, to assess differences between Inoculated and Control microcosms, for plants harvested at 4 weeks, and plants harvested at 6 weeks.

## Results

### Seedlings before test and synthesis of ectomycorrhizas

**Figure 4.**
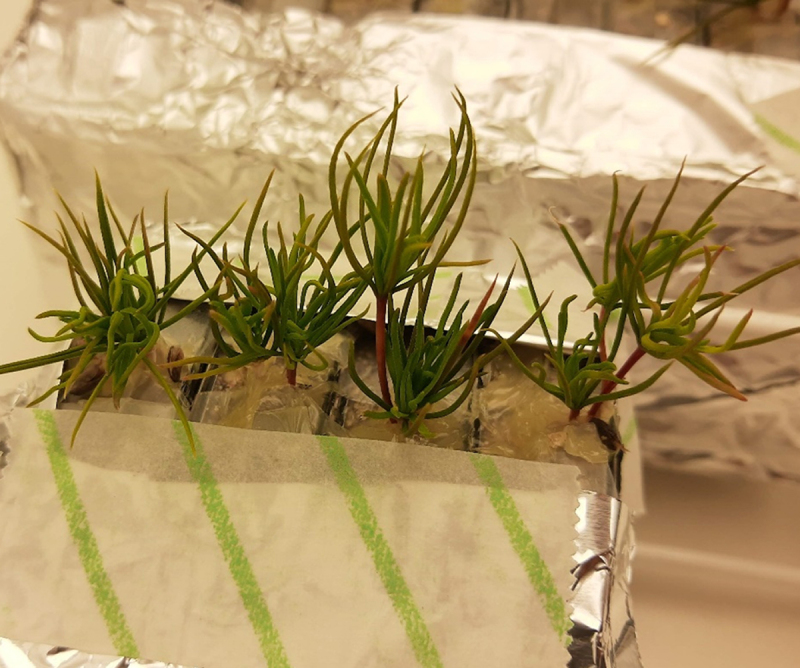
Pine seedlings in vertical petri dishes shortly before being harvested and transplanted to the microcosms, showing signs of stress.

After two weeks in the Petri dishes, the needles of a few seedlings started to become chlorotic (Figure 4). When the Petri dishes were opened, the substrate looked dry, despite visible moisture condensed in the walls of the dishes. Nevertheless, the analysis of the seedlings not used in the microcosm showed that 88% of them (*n* = 41) presented fine root tips surrounded by hyphae, which we used as an indication of ectomycorrhizas partially or completely formed (Figure 3c).

**Figure 5.**
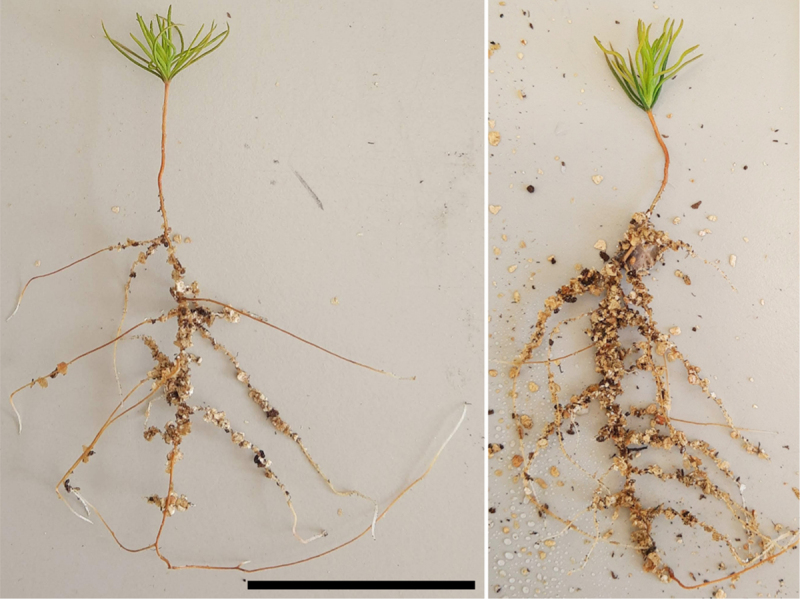
Seedlings of *P. sylvestris* 4 weeks after inoculation with *S. granulatus* in Petri dishes with peat and vermiculite, before being used in the microcosms. On the left, a control seedling. On the right, an inoculated seedling. Note the unusually extensive development of the root system. The scale bars represent 5 cm for both seedlings.

After inoculation in the Petri dishes, both inoculated and control seedlings had unexpectedly extensive root growth (Figure 5). All root morphological features that were evaluated are presented in Table 1. Seedlings from the inoculated treatment had higher total root length, increased surface area of roots, and higher number of root tips than control plants.

**Table 1.** Comparison of mean root features (± standard deviation) between seedlings of *P. sylvestris* inoculated with *S. granulatus* (ECM) and control treatments without inoculation (NM). ‘Mycorrhizal structures’ refer to the absolute number of seedlings with at least one root tip with hyphae, therefore the standard deviation is not applicable.

Root traits	NM (*n* = 26)	ECM (*n* = 41)	*R*^*2*^	*F*	*p*	
Length (cm)	48. 9 ± 18.6	62.6 ± 22.2	0.10	6.86		0.011
Surface area (cm^2^)	6.3 ± 2.6	7.7 ± 2.8	0.06	4.18		0.045
Root volume (cm^3^)	0.07 ± 0.03	0.08 ± 0.03	0.03	2.00		0.162
Fine root tips (count)	78.1 ± 30.0	117.7 ± 44.4	0.20	16.0		0.0002
Mycorrhizal structures (count)	—	36	—	—		—

### Microcosms

**Figure 6.**
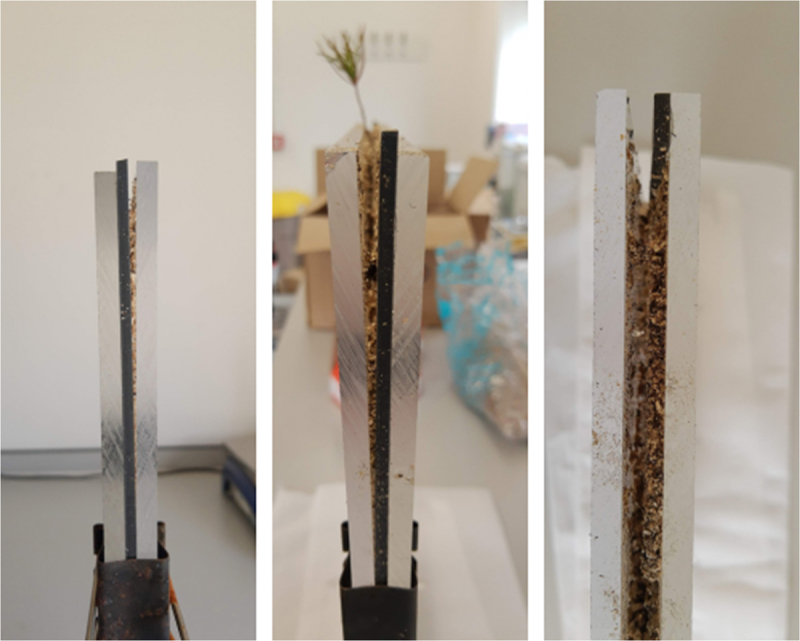
Gaps opened in the microcosms due to the folding back of the Perspex plates. In some cases, the gaps between the plates were as wide as 0.5 cm.

During the course of the experiment (approximately four days after setting up the microcosms), the microcosms started bending outwards (Figure 6). This exposed the substrate to become dry in its upper layer, which required adjustments during the experiment, i.e., the inclusion of additional clips and clamps, a refill of substrate, and further water addition.

None of the microcosms exhibited significant hyphal growth like the ones shown in the classical studies we used as a reference (e.g^22–25^). A few roots grew into the mazes but did not touch the bottom. It was impossible to quantify the biomass inside the maze because the roots attached to the Perspex, and the whole root system had to be moved when opening the microcosms. To try a method for solving this problem, we tested freezing two microcosms in a −20 °C cold room before opening them. This held the roots in place, although it broke them as they became brittle. With this method, precision with the WinRHIZO measurements is lost, but at least it presumably allows quantifying the biomass inside and outside of the U-shaped mazes.

### Growth parameters after 4 and 6 weeks

The results for all the parameters assessed after 4 and 6 weeks after the experiment are presented in Table 2. No significant difference was observed in any of the parameters between treatments after 4 or 6 weeks. All the raw data for the seedlings before and after the microcosm tests can be found in the Supplementary Material.

**Table 2.** Root and shoot features of *P. sylvestris* seedlings inoculated with *S. granulatus* (ECM) and non-inoculated control (NM) after 4 and 6 weeks (values are averages ± standard deviation). The one-way ANOVA test did not show statistically significant differences (*p* ≤0.05) between treatments, regardless of the growth period. Note that root tips means the total number of fine root tips defined by WinRHIZO, and not necessarily colonised root tips.

	4 weeks					6 weeks				
Parameter	NM (*n* = 8)	ECM (*n* = 8)	*R*^*2*^	*F*	*p*	NM (*n* = 7)	ECM (*n* = 7)	*R*^*2*^	*F*	*p*
Root length (cm)	140.2 ± 34.9	127.2 ± 23.2	0.04	0.60	0.45	158.3 ± 36.1	177.0 ± 70.9	0.02	0.24	0.63
Root surface area (cm^2^)	17.8 ± 5.2	16.0 ± 2.3	0.04	0.56	0.47	18.3 ± 4.7	21.0 ± 9.0	0.02	0.31	0.59
Root total volume (cm^3^)	0.2 ± 0.1	0.2 ± 0.0	0.03	0.49	0.50	0.2 ± 0.0	0.2 ± 0.1	0.03	0.37	0.56
Root tips (count)	336.7 ± 135.0	270.6 ± 58.5	0.10	1.58	0.23	380.3 ± 58.2	406.7 ± 147.5	0.01	0.08	0.78
Root mass (g)	0.04 ± 0.01	0.04 ± 0.01	0.02	0.26	0.62	0.05 ± 0.02	0.06 ± 0.02	0.04	0.51	0.49
Shoot mass (g)	0.05 ± 0.02	0.04 ± 0.01	0.02	0.36	0.56	0.05 ± 0.02	0.06 ± 0.02	0.01	0.13	0.72
Total mass (g)	0.09 ± 0.03	0.08 ± 0.02	0.03	0.37	0.55	0.10 ± 0.03	0.11 ± 0.04	0.03	0.32	0.58
Shoot height (cm)	2.0 ± 0.6	1.6 ± 0.7	0.06	0.87	0.37	2.0 ± 0.6	1.6 ± 0.6	0.05	0.62	0.45

## Discussion

In this work, we hypothesised that in a microcosm setting, hyphae of ectomycorrhizal fungi would grow faster than the roots of their host plant and guide the growth of these roots, preventing them from being trapped inside a U-shaped maze placed below the seedlings. The seedlings were harvested after growing for 4 and 6 weeks but, due to technical issues, it was not possible to test this hypothesis.

The experiment was unlikely to succeed when, 8 weeks after inoculating *P. sylvestris* seedlings with the fungus *S. granulatus*, they presented enormous root growth. This was unexpected, as we followed similar protocols to classical works of the past, in particular^22–25^. In these experiments, the initial root growth was minimal, rarely exhibiting more than two lateral roots. This amount of lateral roots would have been ideal for testing our hypothesis in the microcosm experiment.

When we opened the Petri dishes where the seedlings were inoculated, we noticed that the substrate looked dry despite condensation in the walls of the dish. Although drought stress is known to increase root growth in many plant species,^30^ drought-stressed *P. sylvestris* actually reduce root growth.^31,32^ Therefore, it is unlikely that the lack of growth medium caused the excessive root growth. A possible reason for such growth could be the genetics of the plant. Different genotypes can yield different growth rates, so perhaps the seedlings from this batch of seeds naturally grew longer roots. Additionally, these seeds were collected from two different locations in the United Kingdom (Shropshire and Norfolk), and obtained through open pollination in the plantations (Chiltern Seeds, *personal communication*). Consequently, the varied genetic of the seeds could lead to high variance in the results, which potentially interferes in how easily the results can be reproduced. In this case, when possible, it would be ideal to use clone seedlings, or at least seeds from the same parent tree, so that at least 50% of the genome of the seedlings is identical.

We acknowledge that this study could have benefitted from more preliminary tests to assess root growth or other potential issues prior to the experiment. However, this work was conceived and conducted as an exploratory, proof-of-concept study. We aimed to assess whether the existing methodology, which has been widely used and reported in the literature (e.g.,^22,23,25^), could be replicated and adapted to a novel hypothesis. Nonetheless, the unexpectedly vigorous root growth observed here highlights the necessity of such preliminary testing in future implementations of this technique. In this case, a researcher willing to use the same technique must be mindful of the time frame required to do all the tests prior to commencement of the actual experiment, for as we have noted, it takes quite a long time from sowing the seeds to having the seedlings inoculated and ready for experimentation.

Ectomycorrhizas were established at a very good rate (up to 88%), which is a good indicator of the vigour and viability of the inoculum. Ensuring inoculum viability is an important step in mycorrhizal research because some ectomycorrhizal fungi stop forming ectomycorrhizal tips after being kept in culture for a long time. However, in our experiment, most colonisation occurred close to the soil surface rather than on newly forming root tips on lateral roots down the soil profile. As such, their positioning would be ineffective to guide root growth. Extensive hyphal growth, like those shown in the classical studies, which was anticipated and considered critical for testing the hypothesis, was not observed.

Problems with this experiment were further aggravated when the Perspex plates started to bend outwards. This exposed the plant roots, dried the substrate, and likely hindered fungal development. This might explain why they did not develop hyphae like in the classical studies, and likely explains why we did not observe any significant difference between the control and inoculated plants. One positive aspect of the procedure was that we did not observe significant levels of contamination despite all these problems, which is very positive for follow-up tests to be done in the future.

Despite this experiment not yielding the output expected, we feel it is important to report it because the information in the material and methods of older papers is often insufficient to allow an accurate replication of the experiments they describe. Here, we synthesised the methods of several papers together and, with information kindly provided by some of the authors (Roger Finlay and Anna Rosling, *personal communication*), we came up with a methodology that represented an ‘average’ of what was done in the past for the studies we used as a reference. Even if the methods employed here did not work completely, we believe it is a step forward for designing a methodology to conduct these types of studies on mycorrhizas. We hope that researchers willing to do similar experiments can learn from our failures and successes and perfect this method, and we urge them to report their methodology with as much accuracy as possible. The technique of synthesising mycorrhizas and growing inoculated seedlings in microcosms is old, but can still provide valuable information about the ecology and behaviour of mycorrhizas and their importance for seedling development and root architecture.

We conclude this report with the following recommendations to anyone interested in using this technique:

- Although it was not a concern in our experiment, it is important to ensure when initiating the experiment that both the seeds and fungal inoculum are fresh and active. Fungal strains that have been in culture too long may prove difficult to inoculate onto seedlings.
- When inoculating the seedlings with mycorrhizal fungi, agar plugs without fungi should be included in the substrate of the control plants as well. This will help to control for differences in the growth of the seedlings due to the agar acting as a source of nutrients.
- Ensure that the Perspex plates are firmly held along the entire length to avoid bending and exposure of the substrate, roots, and agar.
- Root growth in our experiment was highly unusual compared to previous microcosm experiments and should be investigated. It may be because of the plant genetics, but we recommend testing seedling growth with and without ectomycorrhizal fungi in different combinations of substrate before initiating further experiments (e.g., different proportions of peat and vermiculite; 1:2, 1:1, 1:0, 2:1, 4:1, 0:1) and also trying different concentrations of MMN medium. For this kind of experiment, ideally, there should be no more than two lateral roots before transplanting to the microcosms.
- Whenever possible, use clones for the seedlings or seeds from the same parent tree to minimise genetic variability and facilitate reproducibility of the results.
- We found that the roots of our seedlings attached to the Perspex plates, making it impossible to open the microcosm without disrupting the position of the roots. Freezing the microcosms at −20 °C before opening them allowed us to recover the biomass inside and outside of the mazes more reliably.
- In our experience, the length of time required to conduct this experiment was too long to allow reasonable adjustments and repetition (>15 weeks before obtaining the data). We highly recommend experimental examination of alternative substrate mixtures in smaller microcosms over shorter time periods before embarking on experiments on the same scale as ours, unless time is not a limiting factor.

Mycorrhizas are among the most widespread terrestrial symbioses in the world, and so much is still unknown about them. Despite falling considerably out of fashion, classical experiments with microcosms can still provide important information about the ecology and behaviour of plants associated with ectomycorrhizal fungi at a relatively low cost. We hope this report inspires researchers to investigate how ectomycorrhizas may influence host plant root growth by improving upon this technique.

## Supplementary Material

Supplementary material.docx

Raw Data Supplementary Material.xlsx
