# Supplementary material for: The pitfalls of ectomycorrhizal microcosms: lessons learnt for future success
Source: Plant Signal Behav. 2025 Jul 7;20(1):2527378. doi: 10.1080/15592324.2025.2527378 (PMC12239769; doi:10.1080/15592324.2025.2527378)
Supplement: Supplementary material.docx [file KPSB_A_2527378_SM8574.docx]

|  | | | | | | | | |
| --- | --- | --- | --- | --- | --- | --- | --- | --- |
| Reference | **Plant species** | **Peat : vermiculite** | **Sterilisation** | **Moistened with** | **Light day/night** | **Irradiance** | **Temperature day/night** | **Time to form association** |
| Rosling et al. 2004 | *Pinus sylvestris* | 1 : 4 |  | MMN |  | 300 µmol PAR | 14-16/6-8 | 8 weeks |
| Finlay and Read 1986 | *Pinus sylvestris* | 1 : 4 | Autoclaved | MMN | 16/8 | 38 W m-² | 15/10 | 8 weeks |
| Duddridge 1986 | *Pinus sylvestris* | 1 : 4 |  | 1 : 4 MMN no sugar : water | 16/8 | 160 µmol PAR | 15/10 |  |
| Bending and Read 1995 | *Pinus sylvestris* | 1 : 3 | Autoclaved | 2 : 1 MMN : water | 16/8 | 150 µmol PAR | 15/10 | 8 weeks |
| Finlay 1989 | *Pinus sylvestris* |  |  | MMN 1.25 g L^-^¹ glucose, 5 g L^-^¹ malt extract | 16/8 |  | 20/15 | 4-9 weeks |

## Supplementary Material 1: Comparison of methods for mycorrhizal synthesis in different papers

This table summarises how different authors synthesised mycorrhizas on *Pinus sylvestris*. Blank cells means that this information could not be found in the paper.

## Supplementary Material 2: Microcosm design

The maze was placed 15.5 cm above the bottom of the microcosm, and 6.5 cm to the sides. Each square has 1 cm side. Blue areas indicate the silicone spacers and the maze.
